# Transient psychosis due to painless thyroiditis in a patient with anxiety disorder: a case report

**DOI:** 10.1186/1752-1947-5-534

**Published:** 2011-10-31

**Authors:** Nobuyuki Kobayashi, Junichi Tajiri, Masahiro Takano

**Affiliations:** 1 Department of Psychosomatic Medicine, Takano Hospital, 4-2-88 Obiyama, Kumamoto, 862-0924, Japan; 2 Tajiri Clinic, 2-6-3 Suizenji, Kumamoto, 862-0950, Japan; 3 Coloproctology Center, Takano Hospital, 4-2-88 Obiyama, Kumamoto, 862-0924, Japan

## Abstract

**Introduction:**

There are few reports on thyrotoxic psychosis caused by diseases other than Graves' disease or toxic nodular goiter.

**Case presentation:**

A 64-year-old Japanese woman was treated for anxiety disorder in our clinic for 10 years. She had five episodes of transient psychosis during the first five years. When she developed psychosis without neck pain 10 years after her first visit, a laboratory reexamination revealed that she had subclinical hyperthyroidism, and tested positive for antithyroid autoantibodies, negative for thyroid stimulating hormone receptor antibody and had decreased radioactive iodine uptake. She was diagnosed as having painless thyroiditis. The hyperthyroidism disappeared within a month, and the psychosis lasted for three months.

**Conclusion:**

To the best of our knowledge, this is the first report of psychosis due to painless thyroiditis-induced hyperthyroidism. Physical symptoms of painless thyroiditis are often so mild that careful differential diagnosis is necessary in the cases of transient psychosis.

## Introduction

Hyperthyroid patients frequently develop mental symptoms [1,2], such as anxiety, irritability, unstable mood, fatigue and insomnia. However, psychosis is a rare complication [3,4]. Many cases of psychosis that were induced by thyrotoxicosis, or thyrotoxic psychosis, were caused by Graves' disease or a toxic nodular goiter. Although destructive thyroiditis is the next most common cause of hyperthyroidism, only one case each of postpartum thyroiditis [5] and subacute thyroiditis [6] have been reported to induce psychosis.

We report on a patient with anxiety disorder who experienced transient psychosis due to the development of painless thyroiditis. The self-limited time course of the psychosis in the patient reported here was similar to that of painless thyroiditis.

## Case presentation

Our patient was a 64-year-old Japanese housewife who was treated for anxiety disorder at our psychosomatic clinic for 10 years. In March of the 11^th ^year after her first visit, she felt dizziness, but no neck pain. She developed the abnormal thought that someone might tell her of past failures, and this idea grew rapidly into delusions and hallucinations. She reported that she often left her home and sought someone who told her about her failures. She complained of sleeplessness but not of a depressive or manic mood. Although she denied our advice to admit herself, she underwent a thorough medical workup.

Our patient's medical history was significant for diagnoses of Hashimoto's thyroiditis, hypertension and hyperlipidemia at the age of 48. At the age of 54, she had consulted our clinic because of dry oral sensation, palpitations, sweating, anxiety and sleep disturbances. Her thyroid function was normal at that time, with serum thyroid stimulating hormone (TSH) levels of 1.8 μIU/mL (normal range, 0.4 μIU/mL to 4.0 μIU/mL) and free thyroxine (fT4) levels of 1.25 ng/dL (normal range, 0.8 ng/dL to 1.9 ng/dL). Serological examination by passive hemagglutination showed antimicrosomal antibody (MCHA) levels of 400 (normal range < 100) and antithyroglobulin antibodies (TGHA) levels of less than 100 (within normal range). She was diagnosed as having generalized anxiety disorder and was treated with supportive psychotherapy and anxiolytics (alprazolam, 1.2 mg). She continued to complain of various anxieties after a slight improvement in her fear for her health.

Our patient had five episodes of delusions and hallucinations, which lasted for one to three months each and occurred once a year for the first five years after her first visit. These episodes were diagnosed as a psychotic disorder, not otherwise specified, and she was treated with haloperidol during the episodes. After her fourth episode, she was prescribed a low dose of haloperidol in order to prevent another episode. Her thyroid function test was reexamined after the fifth episode and found to be normal.

At reexamination, her consciousness was alert, and convulsion or myoclonus was not observed. She politely complained that she heard someone telling her about failures, for example, "She had spoken ill to her neighbor," and she felt regret for her past faults. She had little insight into her abnormal thoughts, but she easily agreed to further medical examination. No psychomotor agitation, mood swing, or disruption of thought was observed. A physical examination showed that her blood pressure was 130/90 mmHg and that her small goiter was palpable. Laboratory data (Table 1) showed negative C-reactive protein (CRP) levels, low serum TSH levels, and normal free triiodothyronine (fT3) and fT4 levels. Tests were positive for thyroid autoantibodies, including MCHA, TGHA, antithyroglobulin antibody (anti-Tg) and thyroid peroxidase antibody (anti-TPO) (both measured by radioimmunoassay), while TSH receptor antibody (TRAb) was negative. Under an iodine-unrestricted diet, radioactive iodine uptake (RAIU) was immediately measured and found to be low: 1.8% after one hour and 2.2% after one and a half hours (normal range: see Discussion). Ultrasonography revealed a diffuse goiter and a left lower parathyroid cyst. A brain computed tomography scan was normal.

**Table 1:** Laboratory data on the onset of the psychotic episode 10 years after the first visit.

	Value	Normal range
TSH (μIU/mL)	0.028	0.4 to 4.0
fT3 (pg/mL)	2.85	2.5 to 4.3
fT4 (ng/dL)	1.7	0.8 to 1.9
MCHA	1600	< 100
TGHA	6400	< 100
anti-Tg (U/mL)	40.5	< 0.3
anti-TPO (IU/mL)	78	< 16
TRAb (%)	15.3	< 20
CRP (mg/dL)	0.1	< 0.3
RAIU (%)	1.8 (1 h) → 2.2 (1.5 h)	See text
Ultrasonography	Diffuse goiter, left lower parathyroid cyst	

**Figure 1:**
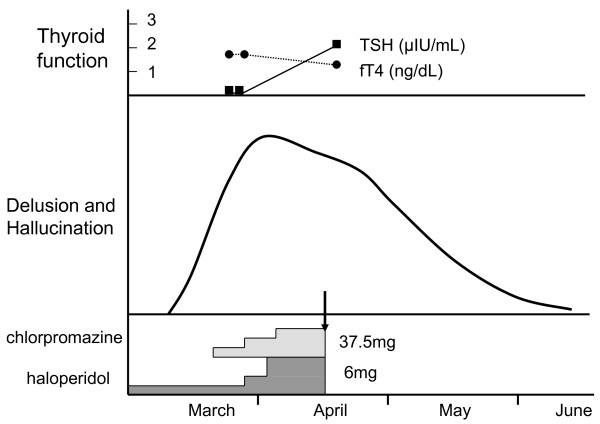
**Course of the psychotic episode that occurred 10 years after the first visit**. Closed squares show serum thyroid stimulating hormone (TSH) levels and closed circles show serum free thyroxine (fT4) levels. The arrow indicates the time of our patient's refusal of drugs.

These results definitively suggested painless thyroiditis. Antithyroid drugs were not delivered, while the dose of haloperidol was increased to 6 mg. Chlorpromazine (37.5 mg) was added, but she refused to take all of the psychotropic medications. In April, her serum TSH (2.07 μIU/mL) and fT4 (1.37 ng/dL) levels returned to normal. Her delusions weakened and disappeared in June (Figure 1).

No delusions or thyrotoxicosis developed for four years after the last delusional episode (14 years after her first visit). Her generalized anxiety disorder recovered without any psychotropic agents.

## Discussion

Painless thyroiditis consists of self-limited thyrotoxicosis. It follows transient hypothyroidism in some cases and occurs during a course of Hashimoto's thyroiditis or Graves' disease in remission. The hyperthyroidism in this disease is usually mild and is often unnoticed. At first, we thought that the delusions in our patient were psychologically induced, and a diagnosis of psychotic disorder, not otherwise specified, was made according to the Diagnostic and Statistical Manual of Mental Disorders, Fourth edition, Text Revision (DSM-IV-TR) [7]. We carefully differentiated from affective disorders, schizoaffective disorder, delusional disorder or schizophreniform disorder. A depressive mood was never a prominent symptom, and there was no effect with the attempted medication of 150 mg of fluvoxamine for three months (data not shown). However, the repeated and self-limited time course of her psychosis was similar to that of painless thyroiditis. Immediate examination after the onset of the delusions was necessary in order to show that the transient psychosis was induced by painless thyroiditis. It was difficult to obtain a definite diagnosis because of the lack of neck pain and the resemblance of the mental symptoms to those seen in anxiety disorder and hyperthyroidism.

In earlier studies, 10% to 20% of thyroid patients were mentally ill [1], and about 1% of thyrotoxic patients developed psychosis [3,4]. These reports were not based on current psychiatric criteria according to the DSM-IV-TR, and the accurate incidence is unclear. There is no specific psychiatric picture of the psychosis that is associated with thyrotoxicosis. One study reported that 14 out of 18 patients with thyrotoxicosis had affective psychoses [8]. Affective psychosis in this report may correspond to schizoaffective disorder in the DSM-IV-TR, which features psychosis that is accompanied by depression or mania. Our case was different from this condition as depression was only a minor symptom.

Although destructive thyroiditis induces self-limited hyperthyroidism, there have been only two case reports of psychosis induced by destructive thyroiditis. In 1998, Bokhari *et al*. reported a case with postpartum thyroiditis and psychosis [5]. Because postpartum psychosis is a common disease, thyroiditis and psychosis may have co-occurred by chance. Currently, postpartum thyroiditis is thought to be one form of painless thyroiditis that develops after delivery [9]. Rizvi discussed a case of thyrotoxic psychosis that was associated with subacute thyroiditis [6]. To the best of our knowledge, this is the first report of thyrotoxic psychosis associated with painless thyroiditis.

One of the key findings of painless thyroiditis is lowered RAIU. The standard method of the measurement of RAIU is to measure radioactive levels 24 hours after radioactive drug intake under an iodine-restricted diet. Immediate measurement is difficult, especially for psychotic patients. One of the authors of this paper developed a simple and immediate method to differentiate painless thyroiditis from Graves' disease [10]. RAIU was measured twice, one hour and one and a half hours after intake under an iodine-unrestricted diet. If the difference between these two values was less than 1%, this author reported that 83.6% of the patients with suppressed TSH had painless thyroiditis. In the present case, the difference in the RAIU values (2.2% and 1.8%) was 0.4% and, hence, painless thyroiditis was highly suspected. This diagnosis was confirmed by the normalization of the subclinical hyperthyroidism one month after onset and by the autoimmune antibodies that were compatible with chronic thyroiditis and negative TRAb.

It remains to be elucidated how the dysfunction of thyroid hormones causes psychiatric symptoms. The mental state would be influenced by the accelerated metabolism of bioamines and interaction with many neurotransmitter systems in a thyrotoxic state. However, this does not sufficiently explain the psychotic symptoms in our patient. Her recovery from psychosis was delayed compared to the hyperthyroidism. Similarly, there was a case report where psychosis developed after the immediate normalization of thyrotoxicosis by intensive care in a patient with Graves' disease [11]. These discrepancies in the time course suggest that psychosis is not a simple result of hyperthyroidism. Moreover, the content of the delusions and hallucinations in our patient was similar to that of the anxiety or fear experienced by the patient before the psychosis. There might have been vulnerability for psychosis, and the hyperthyroidism may have only acted as a transient promoting factor. For other psychiatric disorders, similar relationships have been reported; anxiety disorder and depression lasted after recovery from hyperthyroidism [12] and other factors may have been involved in the mental disorders associated with hyperthyroidism. One possible explanation is that antithyroid antibodies may play some role in psychiatric symptoms, irrespective of hyperthyroidism. There are reports associating thyroid autoimmunity with anxiety disorders [13] and acute psychosis in the presence of more or less prominent affective and/or neurological symptoms [14]. Brain perfusion abnormalities have been found in patients with euthyroid autoimmune thyroiditis [15], which demonstrates another possibility of the involvement of thyroid antibodies in the central nervous system.

## Conclusion

To the best of our knowledge, this is the first report of psychosis due to painless thyroiditis-induced hyperthyroidism. Physical concerns of painless thyroiditis are often so mild that careful differential diagnosis is necessary in the case of psychosis with thyroid disease and especially for chronic thyroiditis.

## Consent

Written informed consent was obtained from the patient for publication of this case report and any accompanying images. A copy of the written consent is available for review by the Editor-in-Chief of this journal.

## Competing interests

The authors declare that they have no competing interests.

## Authors' contributions

NK took part in the treatment of the patient and drafted the manuscript. JT took part in the treatment of painless thyroiditis. MT provided instruction and advice on the treatment strategy. All authors read and approved the final manuscript.

## References

[B1] Bursten B Psychoses associated with thyrotoxicosis Arch Gen Psychiatry 1961 4 267 273 13689243 10.1001/archpsyc.1961.01710090053007

[B2] Placidi GP Boldrini M Patronelli A Fiore E Chiovato L Perugi G Marazziti D Prevalence of psychiatric disorders in thyroid diseased patients Neuropsychobiology 1998 38 222 225 10.1159/000026545 9813461

[B3] Brownlie BEW Wells JE The epidemiology of thyrotoxicosis in New Zealand: incidence and geographical distribution in North Canterbury 1983-1985 Clin Endocrinol 1990 33 249 259 10.1111/j.1365-2265.1990.tb00489.x 2225482

[B4] Gagliardi JP Clary GL Treatment of thyrotoxicosis-induced psychosis Psychopharmacol Bull 2002 36 7 13 12858137

[B5] Bokhari R Bhatara VS Bandettini F McMillin JM Postpartum psychosis and postpartum thyroiditis Psychoneuroendocrinology 1998 23 643 650 10.1016/S0306-4530(98)00034-1 9802134

[B6] Rizvi AA "Thyrotoxic psychosis" associated with subacute thyroiditis South Med J 2007 100 837 840 10.1097/SMJ.0b013e318063ed4e 17713314

[B7] American Psychiatric Association Diagnostic and statistical manual of mental disorders 2000 4 Washington DC; American Psychiatric Association Text Revision

[B8] Brownlie BEW Rae AM Walshe JWB Wells JE Psychoses associated with thyrotoxicosis-'thyrotoxic psychosis.' A report of 18 cases, with statistical analysis of incidence Eur J Endocrinol 2000 142 438 444 10.1530/eje.0.1420438 10802519

[B9] Felicetta JV Painful, painless, and postpartum thyroiditis. Distinct entities or merely variants? Postgrad Med 1989 86 269 272 2678067 10.1080/00325481.1989.11704451

[B10] Tajiri J Radioactive iodine uptake test: Is it possible to differentiate painless thyroiditis from Graves' disease within 1.5 hours in the shortest cases? Nihon Naibunpi Gakkai Zasshi 2004 80 126 Abstract in Japanese

[B11] Irwin R Ellis PM Delahunt J Psychosis following acute alteration of thyroid status Aust N Z J Psychiatry 1997 31 762 764 10.3109/00048679709062692 9400884

[B12] Bunevicius R Velickiene D Prange AJ Jr Mood and anxiety disorders in women with treated hyperthyroidism and ophthalmopathy caused by Graves' disease Gen Hosp Psychiatry 2005 27 133 139 10.1016/j.genhosppsych.2004.10.002 15763125

[B13] Carta MG Loviselli A Hardoy MC Massa S Cadeddu M Sardu C Carpiniello B Dell'Osso L Mariotti S The link between thyroid autoimmunity (antithyroid peroxidase autoantibodies) with anxiety and mood disorders in the community: a field of interest for public health in the future BMC Psychiatry 2004 4 25 10.1186/1471-244X-4-25 15317653 PMC516779

[B14] Wilcox RA To T Koukourou A Frasca J Hashimoto's encephalopathy masquerading as acute psychosis J Clin Neurosci 2008 15 1301 1304 10.1016/j.jocn.2006.10.019 18313925

[B15] Piga M Serra A Deiana L Loi GL Satta L Di Liberto M Mariotti S Brain perfusion abnormalities in patients with euthyroid autoimmune thyroiditis Eur J Nucl Med Mol Imaging 2004 31 1639 1644 10.1007/s00259-004-1625-7 15290119

